# Referral to pediatric gastroenterology for chronic abdominal pain differs by patients' individual, family, and community characteristics

**DOI:** 10.1002/jpn3.70185

**Published:** 2025-08-12

**Authors:** Shaunte McKay, John E. Fortunato, Stella W. Karuri, Bonnie S. Essner

**Affiliations:** ^1^ Division of Pediatric Gastroenterology, Hepatology and Nutrition, Emory University School of Medicine Children's Healthcare of Atlanta Atlanta Georgia USA; ^2^ Division of Gastroenterology, Hepatology, and Nutrition Ann & Robert H. Lurie Children's Hospital of Chicago Chicago Illinois USA; ^3^ Department of Pediatrics Northwestern University Feinberg School of Medicine Chicago Illinois USA; ^4^ Stanley Manne Children's Research Institute Chicago Illinois USA; ^5^ Division of Gastroenterology, Hepatology, and Nutrition, Pritzker Department of Psychiatry and Behavioral Health Ann & Robert H. Lurie Children's Hospital of Chicago Chicago Illinois USA; ^6^ Department of Psychiatry and Behavioral Sciences Northwestern University Feinberg School of Medicine Chicago Illinois USA

**Keywords:** disorders of gut–brain interaction, health disparities, healthcare access, inequities

## Abstract

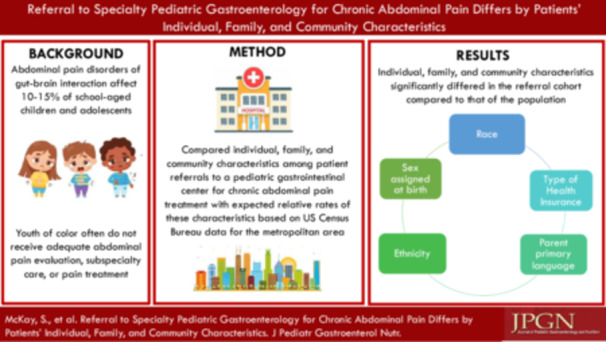

## 1 INTRODUCTION

Abdominal pain disorders of gut–brain interaction (AP‐DGBIs) are prevalent, with approximately 10%–15% of school‐aged children meeting criteria for at least one of these conditions.^1^ Biopsychosocial factors contribute to visceral and somatic symptoms and functional disability in patients with AP‐DGBIs.^2^ Persistent abdominal pain is associated with a higher risk of co‐occurring mental health conditions. Thus, a multidisciplinary care approach utilizing a primary care provider partnered with a pediatric gastroenterologist, pain specialist, and behavioral health professional can reduce healthcare utilization and improve care for DGBI patients.^3^

Inequities in healthcare for historically marginalized racial and ethnic communities are well known.^4^, ^5^ There is evidence that youth of color do not receive adequate abdominal pain evaluation, subspecialty care, or pain treatment despite presenting with similar rates of abdominal pain as White children.^5^ Additionally, it has been shown through experimental models that Black patients may experience more intense pain and lower pain thresholds compared to non‐Hispanic White patients.^6^ Despite these findings, medical professionals express false beliefs about African‐Americans' pain experiences and provide suboptimal treatment recommendations for Black patients compared to White patients.^7^ False beliefs may therefore affect patient access to effective multidisciplinary pain care. Disproportionate access to healthcare has detrimental effects on patients from marginalized communities and ultimately leads to increased healthcare costs.^8^, ^9^, ^10^ There is limited data on individual, family, and community level factors, as well as healthcare systemic factors, that contribute to multidisciplinary care access for patients with AP‐DGBIs of varying racial and ethnic identities.^9^, ^10^ Black, compared to White, patients receive less subspecialty care for AP‐DGBIs due to a relatively lower rate of referral to tertiary centers. The primary objective of this study was to compare referrals of Black and White patients to a large, urban pediatric gastroenterology program for treatment of AP‐DGBIs against expected referrals based on demographic representation in the Chicago metropolitan area (CMA).^11^, ^12^ We also compared referral rates of patients of other historically minoritized racial and ethnic groups to White patient referrals in the same cohort, and evaluated whether other patient factors (e.g., sex assigned at birth) and family factors (e.g., health insurance type) of the referral sample were significantly different than what would be expected given the broader metropolitan area.

## 2 METHODS

We conducted a retrospective review of patient referrals to our tertiary pediatric gastroenterology center from September 1, 2018, to September 1, 2021.

### 2.1 Ethics statement

The Lurie Children's Office of Research Integrity and Compliance approved an Institutional Review Board exemption.

### 2.2 Study participants

Of the patients aged 6–18 years old, 10,866 referrals had International Classification of Diseases (ICD)‐10 diagnostic codes aligned with chronic abdominal pain. We excluded patients <6 and ≥18 years of age, duplicate referrals to our clinics, referrals from pediatric gastroenterologists, and patients with ICD‐10 diagnostic codes for primary inflammatory, structural, and/or motility gastrointestinal diseases. We collected: (1) patient factors: sex assigned at birth, age, race, and ethnicity; (2) family factors: parent primary language, and health insurance type; (3) community factors: zip code of primary residence; and (4) systemic factors: referring clinician type, from medical records and referral databases. We accessed the United States (US) Census Bureau's Adjusted 2020 estimated median family income per zip code and substituted the figure corresponding to a patient referral's zip code as a proxy for that patient's family income.

### 2.3 Statistical analysis

Descriptive statistics were used to characterize the demographics of the referral sample. Chi‐square (*X* ^2^) analyses were used to determine statistically significant differences between sex assigned at birth, race, ethnicity, parent primary language, and insurance type for referrals to pediatric gastroenterology compared to the expected relative rates based on the 2020 US Census data for areas of the CMA in which the majority of medical center patients live.^11^, ^12^ Post hoc exact binomial tests compared racial subgroups of the referral cohort to the respective CMA subgroup to determine between which subgroups there existed a statistically significant difference. Each binomial subgroup analysis compared White patients with patients of one of the other race categories: Black/African American, Asian, Multiple Races, and Other. The Bonferroni method was used to adjust for multiplicity, for an overall significance level of 0.05, and each subgroup comparison was tested at a significance level of 0.125.

## 3 RESULTS

After applying the exclusion criteria, 3843 referrals were included in our analyses (mean age = 2 years, standard deviation = 3). Characteristics of these referrals were compared to those of the CMA population of 1,591,855. See Table 1 for demographic descriptive characteristics of the referral cohort and CMA population. There was a significant difference in sex assigned at birth (*X* ^2^ = 272, *p* < 0.001), race (*X* ^2^ = 1096, *p* < 0.001), Hispanic versus non‐Hispanic ethnicity (*X* ^2^ = 49, *p* < 0.001), primary household language (*X* ^2^ = 562, *p* < 0.001), and health insurance type (*X* ^2^ = 139, *p* < 0.001) in the cohort of patients referred for subspecialty gastrointestinal care compared to the expected proportion of patients with those demographic characteristics in the CMA population (Table 1). Male‐identifying patients were referred at a significantly lower percentage (37.6%); female‐identifying patients were referred at a significantly higher percentage (62.4%) than the expected proportion of patient referrals of each sex‐assigned‐at‐birth group based on the surrounding CMA reference population. Patients from English‐speaking households were referred at a significantly higher percentage (88.0%), and patients from Spanish‐speaking homes were referred at a significantly lower percentage (11.0%). Patients with private insurance were referred at a higher percentage (64.3%), and patients with Medicaid were referred at a lower percentage (35.5%). Similarly, patients who identified as Hispanic were referred at a significantly lower percentage (25.4%), and non‐Hispanic patients were referred at a significantly higher percentage (71.6%) as compared to the reference population. Post hoc exact binomial subgroup tests revealed that the proportion of Black patients in the Black and White referral cohort subgroup was significantly less (0.08) than the comparable proportion in the CMA population (0.23) (Table 2). Similarly, the proportion of patients in the referral cohort who identified as Asian American (0.05) and as multi‐racial (0.04) in those respective subgroup analyses was significantly less than the comparable proportions in the CMA population (0.09 and 0.12, respectively). The proportion of patients whose race was categorized as “other” (0.28) was significantly greater than the comparable proportion in the CMA population (0.16). The average median income per zip code in the referral sample was $113,811, compared to $90,760 in the CMA. Additionally, the majority of referrals (82.5%) were from pediatric primary care physicians.

**Table 1:** Abdominal pain‐disorders of gut‐brain interaction referral cohort versus Chicago metropolitan area census data.

Individual factors	Patient referrals cohort		Chicago metropolitan census (5–17)		*X* ^2^, *p*
	*N* = 3843	% = 100	*N* = 1,591,855	% = 100	
Age (years)	12 ± 3				
Sex assigned at birth					
Male	1444	37.6%	810,604	50.9%	272, <0.001
Female	2399	62.4%	781,242	49.1%	
Race					
White	2443	63.6%	918,304	57.7%	
Black/African American	223	5.8%	274,912	17.3%	
Asian American	138	3.6%	92,894	5.8%	1096, <0.001
Multiple races	95	2.5%	125,866	8.0%	
Other^a^	944	24.6%	179,879	11.3%	
Ethnicity					
Hispanic	977	25.4%	502,103	31.5%	
Non‐Hispanic	2752	71.6%	1,089,752	68.5%	49, <0.001
Other^b^	114	2.9%		n/a	

Family factors	Patient referrals cohort		Chicago metropolitan census (5–17)		X^2^, *p*
Household language					
English	3384	88.0%	1,180,745	71.4%	562, <0.001
Spanish	422	11.0%	336,260	20.3%	
Other	37	1.0%	136,133	8.3%	

	Patient referrals cohort		Chicago metropolitan census (6–18)		*X* ^2^, *p*
	*N* = 3843	% = 100	*N* = 1,606,007	% = 100	
Insurance type					
Private	2471	64.3%	1,037,571	64.6%	
Medicaid	1364	35.5%	511,495	31.8%	139, <0.001
Other^c^	8	0.2%	56,941	3.6%	

a Native American, Alaskan Native, Native Hawaiian, and Pacific Islander are included in this category.

b Unknown or patients who declined to identify ethnicity are included in this category.

c Self‐pay and hospital‐based financial assistance are included in this category.

**Table 2:** Post hoc exact binomial tests comparing the proportion of non‐White patients in the referral cohort with the proportion of non‐White youth in the Chicago metropolitan area subgroup.

Race subgroup	Cohort subgroup non‐White proportion (1)	Chicago metropolitan area subgroup non‐White proportion (2)	Exact binomial test *p* a
Black/African American and *White*	0.08	0.23	<0.0125
Asian American and *White*	0.05	0.09	<0.0125
Multiple Races and *White*	0.04	0.12	<0.0125
Other (includes American Indian and Native Hawaiian) and *White*	0.28	0.16	<0.0125

a Hypothesized null: referral subgroup non‐White proportion = Chicago metropolitan area subgroup non‐White proportion.

## 4 DISCUSSION

Findings of our study demonstrated a lower proportion of Black versus White patients were referred for subspecialty AP‐DGBI treatment. Similarly, there were lower proportions of patients of other historically minoritized racial and ethnic groups in the referral cohort subgroups than the proportions of youth with corresponding demographic characteristics in the surrounding urban, metropolitan population. The referral cohort also included a disproportionate representation of private insurance holders, patients from English‐speaking households, and an estimated higher median income compared to the proportions of youth in the CMA with those characteristics. These disproportionate patient, family, and systemic factors identified within our cohort suggest potential disparities in access to pediatric gastroenterology care for AP‐DGBI patients related to racial and ethnic identity and economic advantage.

The study was limited by the data available in our single‐center referral database and the nature of the US Census data. For example, the referral database did not include information on possible individual, environmental, or clinician‐level factors that could potentially affect the clinician's decision to refer patients to subspecialty pediatric gastrointestinal care. Some factors may include clinicians' competing demands, patient refusal of referral placement, and clinician knowledge gaps that may have contributed to disproportionately lower rates of referrals for historically marginalized groups. This single center analysis also could not account for possible referrals placed to healthcare systems outside of our large pediatric gastroenterology network. However, we accounted for the potential that patients with AP‐DGBIs may also be referred to systems outside our network by including only regions of the CMA from which the majority of our system's patients are typically referred, rather than the entire CMA as a reference population. Future studies should consider multi‐center comparisons of individual, family, systemic, and clinician factors that affect referrals for AP‐DGBIs. Due to the small proportions of patients in the referral cohort who identified Native American/Alaskan Native or Native Hawaiian/Pacific Islander, patients in our referral cohort with those racial identities had to be combined with patients who self‐identified as “other” race for Chi‐square analyses. Finally, due to the limitations of the Census data, it was not possible to conduct more nuanced comparisons of the proportions of patients of specified racial and ethnic identities with various family characteristics (e.g., health insurance type) in the referral cohort and the corresponding proportions of patients with those same combinations of racial/ethnic and family characteristics in the CMA reference population. However, via post‐hoc analyses, there are clear indications of disproportionate referrals for youth of color when compared to the White subgroups.

## 5 CONCLUSION

Results from this study provide preliminary evidence for systematic differences in patient referral to pediatric gastroenterology subspecialty care based on patient racial and ethnic identity and other indicators of economic advantage.

## CONFLICT OF INTEREST STATEMENT

The authors declare no conflicts of interest.
